# Fetal Aortic and Umbilical Doppler Flow Velocity Waveforms in Pregnancy: The Concept of Aortoumbilical Column

**DOI:** 10.2174/011573403X255256230919061018

**Published:** 2023-10-10

**Authors:** De Almeida Ana Beatriz, Morais Ana Rita, Ferreira Miguel, Gaio Ana Rita, Guedes-Martins Luís

**Affiliations:** 1 Department of Obstetrics and Gynecology, Centro Hospitalar Universitário de Santo António, University of Oporto, Oporto, Portugal;; 2 Instituto de Ciências Biomédicas Abel Salazar, University of Oporto, Oporto, Portugal;; 3 Department of Mathematics, Faculty of Sciences, University of Oporto, Oporto, Portugal;; 4 Fetal Medicine Centre, Centro Hospitalar Universitário de Santo António, University of Oporto, Oporto, Portugal

**Keywords:** Cardiovascular system, doppler ultrasound, fetal descending aorta, umbilical artery, flow velocity waveform, aortoumbilical column

## Abstract

Low impedance within the uteroplacental circulation is crucial for fetal development. Flow velocity waveforms (FVW) have been established for the aortic and umbilical arteries in low-risk pregnancies during the second half of pregnancy, but data regarding early gestation is limited. Both vascular territories exhibit higher impedance patterns in pregnancies complicated by fetal growth restriction (FGR), hypertensive disorders, fetal anemia, and chromosomal abnormalities. Early identification of these complications is critical in obstetric practice, to reduce perinatal morbidity and mortality through prevention and close antenatal surveillance. Available data suggest that aortic and umbilical impedances follow the same variation pattern as pregnancy progresses. This observation implies that both vessels may be considered as a single artery, referred to as the “aortoumbilical column”. Our hypothesis posits that changes in the hemodynamic pattern of this column could identify high-risk pregnancies, particularly those complicated by preeclampsia, FGR, intrauterine fetal demise, fetal aneuploidies, and fetal anemia. Understanding vascular embryogenesis and the FVWs of the aortic and umbilical arteries enables comprehension of impedance changes throughout normal pregnancies. The continuous variation in impedance along a single vessel supports our concept of the aortoumbilical column. Deviations from the regular pattern could assist in identifying compromised fetuses during early pregnancy. Further research on normal aortoumbilical column FVW and the development of reference charts is necessary to consider this arterial column as a screening tool in clinical practice.

## 1 INTRODUCTION

Fetal development relies on increased fetal cardiac output and the maintenance of umbilical circulation [1]. Blood flow in the fetal descending aorta represents about 75% of cardiac output. Experimental animal studies have demonstrated that aortic blood flow is evenly distributed to the upper and lower parts of the fetal body [1, 2]. In addition to supplying arterial flow to fetal organs, the abdominal section of the fetal descending aorta also provides blood flow to the placental compartment. The umbilical arteries extend to the placental bed, contributing to approximately 50% of the aortic blood flow [1, 3]. Consequently, assessing abdominal aortic blood circulation can be beneficial in monitoring changes in compliance and resistance within both the fetal and placental beds [1-3]. During the late-first and early-second trimesters, significant changes in arterial FVW reflect the physiological reduction of vascular resistance [2, 4]. The introduction of pulsed Doppler ultrasound in obstetrical practice has enabled the evaluation of fetal and uteroplacental circulations [5]. This noninvasive technique has facilitated the establishment of normal FVW in uncomplicated gestations and the detection of hemodynamic changes in high-risk pregnancies [4-6]. Extensive research has been conducted on umbilical circulation due to its clinical relevance in assessing fetal growth and well-being.

Additionally, there is a growing interest in the assessment of fetal aortic Doppler as it is believed to have a strong association with fetal cardiac function and various fetal pathologies, including FGR, hypoxia, and fetal anemia [2, 5].

This review provides a comprehensive summary of the existing knowledge on fetal aortic and umbilical circulations and their Doppler characteristics during pregnancy, encompassing both low-risk and complicated pregnancies. Additionally, we propose a novel concept of the “aortoumbilical column”, which describes the continuous impedance variations observed along the fetal descending aorta and umbilical arteries. Our hypothesis suggests that evaluating impedance changes within the aortoumbilical column could aid in the early identification of high-risk pregnancies, even as early as the first trimester of gestation.

## 2 EMBRYOGENESIS OF THE FETAL VASCULAR SYSTEM

The development of the cardiovascular system initiates when the embryo reaches a length of approximately 0.4 mm, which corresponds to 3 gestational weeks [7, 8]. During early pregnancy, a unique process of cell interactions and differentiation signals commences, aiming to achieve morphological maturity and establish an efficient unit to support the developing fetus [9]. Consequently, the cardiovascular system becomes the first functional organ system in the human embryo [9]. In the early stages of pregnancy, nutrient distribution to embryonic cells primarily occurs through diffusion, which eventually becomes insufficient to ensure proper embryo growth [7, 9]. Therefore, the development of a vascular network becomes crucial, occurring concurrently with cardiac contraction and the circulation of red blood cells [10]. The initial signs of vasculogenesis can be observed in the extraembryonic yolk sac mesoderm, as mesodermal cells form blood islands. These consist of clusters of erythroblasts, and angioblasts, which serve as precursors of erythrocytes and endothelial cells, respectively [9, 10]. Subsequently, two types of vasculogenesis can be described: type I vasculogenesis, in which angioblasts arise at the site of vessel emergence (*e.g.*, the establishment of the dorsal aorta), and type II vasculogenesis, where angioblasts migrate from a distant location and form elongated channels (*e.g.*, development of the ventral aorta) [9, 10]. Gene knockout experiments have demonstrated the significance of vascular endothelial growth factor (VEGF) and its corresponding receptors (VEGFR-2 and VEGFR-1) in embryonic vasculogenesis [9]. However, the formation of a functional vascular network occurs only when angioblastic cords undergo lumenization. Many researchers suggest that this process involves the polarization of endothelial cells within the angioblastic cord, leading to detachment from each other through contractile forces generated by actin-myosin fibers [11]. While not an absolute condition, pressure induced by cardiac activity enhances lumenization and is considered an essential factor in establishing a complete circulatory system [10]. Subsequently, the migration of erythrocytes from blood islands and their circulation within the established vessels expose endothelial cells to shear stress, which promotes arterial and venous differentiation as well as vascular remodeling through gene activation and expression [9].

### 2.1 The Aorta

The dorsal aorta acts as an embryonic precursor of the fetal descending aorta and it originates from cells of the proximal lateral intraembryonic mesoderm [10, 12]. These cells are symmetrically arranged around the body axis and sequentially form the endocardial tubes, the ventral aorta, and the left and right dorsal aorta [9]. While the diameter of these vessels increases significantly during angiogenesis, the primary dorsal aorta undergoes a lateral-to-medial translocation [9, 12]. This translocation enables the fusion of both vessels at the midline, caudally to aortic arches [13]. Subsequently, the dorsal aorta connects anteriorly to the primitive heart *via* the truncus arteriosus, and posteriorly to the vitelline arteries at the umbilicus level, establishing the primary vascular network [12].

The truncus arteriosus and the distal portion of the aortic sac correspond to the embryological precursors of the ascending aorta. The proximal segment of the original aortic arch is formed through septation of these structures around 4 to 5 weeks after conception [14]. The final aortic branches emerge from the aortic arches, which connect the right and left dorsal aorta to the aortic sac. These branches develop concurrently with the pharyngeal arches, through signaling processes induced by neural crest cells stimulated by the pharyngeal arches [13]. For example, PITX2 expression in mesodermal cells of the pharyngeal arches and aortic sac regulates the laterality process, which is important for establishing the original aortic arch later in intrauterine life [13]. In humans, there are six pairs of aortic arches Fig. (**1A**). Pairs I, II, and V regress, while pair VI continues with the primary pulmonary trunk, giving rise to the pulmonary arteries and the ductus arteriosus. The III-aortic arch forms the common carotid arteries, and the left IV-aortic arch develops the segment of the original aortic arch between the left common carotid artery and the ipsilateral subclavian artery [13]. By the end of embryogenesis, the descending aorta develops from the primary dorsal aorta below the T4 level [14].

As different organ systems develop, intersegmental arteries originate from the dorsal aorta to give rise to major vessels in adult circulation [15]. Furthermore, the celiac artery and the superior and inferior mesenteric arteries develop from the vitelline arteries of the yolk sac, connecting to the dorsal aorta at the level of the C7, T2, and T12 vertebrae, respectively [13, 14]. This connection between the dorsal aorta and vitelline arteries (later becoming the umbilical arteries) remains important, as it establishes a link with the placental circulation [13].

The final aorta is then divided into five anatomical segments: the aortic root, ascending aorta, aortic arch, descending aorta, and abdominal aorta Fig. (**1B**) [14, 15]. Understanding the embryogenic development process helps to comprehend the regular establishment of the great vessels and the mechanisms involved in common anomalies of the vascular system. As a result, complex developmental processes primarily occur in the proximal segment of the aorta, explaining the significant number of abnormalities observed at this site during the antenatal period [14].

### 2.2 The Umbilical Arteries

After the establishment of embryonic nutrition through cell diffusion mechanisms, a hemotrophic mode of nutrition becomes dominant. This involves the development of the viteline bloodstream, and later the placental circulation during intrauterine life [16]. These circulations become functional from the onset of the heartbeat until the completion of organogenesis, which typically occurs around 9 weeks of gestation [17]. The yolk sac plays an important role in the early development of the human embryo, serving not only as a transient nutritive structure but also participating in biosynthesis and hematopoiesis processes [16]. It emerges as part of the embryoblast (hypoblast) during the second week of gestation and is situated within the exocelomic cavity [18, 19]. Subsequently, the primary yolk sac regresses, and a secondary yolk sac develops, establishing a connection with the midgut of the developing embryo *via* the vitelline duct [18]. By the beginning of the third week after fertilization, the allantois forms as an outgrowth of the yolk sac into the connecting stalk, giving rise to the primitive umbilical cord [17, 18]. The allantoic and vitelline ducts are fully established by the end of the fourth week [18]. Mesenchymal cells surrounding the extraembryonic mesoderm will then form vascular plexuses within the yolk sac and the allantois, each connecting to the dorsal aorta within the embryo [16, 19, 20]. Consequently, the vitelline and umbilical arteries arise, respectively.

Following an initial period of dominance by vitelline vessels, the umbilical vessels assume the primary circulation in the embryo [18, 20]. Initially, the allantoic vessels give rise to a single umbilical artery, which then bifurcates into two umbilical arteries as the connecting stalk elongates [21, 22]. These two umbilical arteries connect with the paired dorsal aorta in the embryonic pelvis, ultimately corresponding to the internal iliac arteries later in gestation [21, 23]. As the amniotic cavity enlarges, it comes into contact with the chorion around 12 weeks of gestation, resulting in the obliteration of the chorionic cavity [13]. At this stage, the amnion encompasses the embryonic stalks, the vitelline vesicle, and the remaining allantois to form the primitive umbilical cord, which serves as the link between the caudal end of the embryo and the decidua basalis [13, 18, 20].

## 3 FETOPLACENTAL CIRCULATION

A successful pregnancy requires adaptations in maternal hemodynamics and the establishment of a functional circulatory system within the fetus and placenta [22]. The fetal circulatory system, accommodating approximately 10% of the fetus's body weight in blood volume, is characterized by four shunting structures: the ductus venosus, foramen ovale, ductus arteriosus, and umbilical arteries, which facilitate adaptive circulation during intrauterine life [22, 24, 25]. However, these structures typically regress from the fetus to the neonate as the pulmonary system develops to become the primary supplier of oxygen in extrauterine life [24].

Around 10 weeks of gestation, the umbilical circulation assumes the role of the primary source of oxygen and nutrients for the developing fetus, replacing the function of the yolk sac with the placenta [26]. In addition to its transportation function, the processes of vasculogenesis and angiogenesis within the placenta are vital in providing continuous loading resistance to support the developing heart [22, 27, 28]. These processes involve interaction with the maternal environment and are regulated by several angiogenic factors, including VEGF-A, placental growth factor (PlGF), and angiopoietin (ANG) -1/-2, acting through FLT-1 and tyrosine kinase receptor (TIE2) signaling, respectively [22, 27, 29]. These vessels will integrate the developing functional fetal cotyledons, consisting of clusters of fetal vessels perfusing a placental villi segment [27]. Simultaneously, endovascular trophoblast cells invade the distal portion of the uterine arteries around 6 to 8 weeks of gestation.

Consequently, vascular resistance decreases, and well-oxygenated blood flow is conducted across intervillous spaces at the mother's placental side [30, 31]. Hence, the uterine arteries serve as afferent channels for the maternal intervillous bloodstream, while the capillary vessels within the villi, and later the umbilical cord vessels, act as efferent channels for oxygen and nutrient transfer [26, 30, 31].

The umbilical vein carries blood with the highest concentration of substrates and oxygen (about 70-80%) into the fetus, reaching the liver via its intra-abdominal segment [24, 26]. At this point, the blood flow can be directed to the left portal vein or continue through the ductus venosus (DV), which bypasses hepatic circulation and connects directly to the inferior vena cava [26, 32]. In early gestation, most well-oxygenated blood passes through the DV, while a minor portion of the venous blood at the left portal vein is merged with the deoxygenated blood from the splanchnic circulation [32-34]. However, as gestational age progresses, the percentage of blood circulating through the DV decreases, constituting only 18 to 25% of umbilical venous flow near term [35]. Given the unique characteristics of DV, a significant blood flow velocity is achieved, allowing for forward delivery of blood to the right atrium against the cardiac pressure gradient [26, 35].

The blood from the hepatic sinusoids and DV continues through the inferior vena cava and reaches the right atrium, where its oxygen saturation determines its subsequent direction [25]. At this point, two functional circulatory systems are determined: the *via* sinistra and *via* dextra [32]. In the *via* sinistra, a significant fraction of well-oxygenated blood passes through an embryonic interatrial defect to the left heart, which acts as a second shunt of fetal circulation named foramen ovale [36]. This shunted blood accounts for approximately 75% of the total left ventricle output and is then directed to the ascending aorta to supply the coronary and carotid arteries [32, 37]. Oxygen saturation at noble organs is approximately 65%, due to mixing with the deoxygenated blood from the pulmonary veins in the left atrium [25]. As gestational age advances, this right-to-left shunting through the foramen ovale decreases due to the development of pulmonary circulation, contributing to only 50% of ventricular filling by the early third trimester [24, 38]. In contrast, the *via* dextra receives poorly oxygenated blood from the inferior and superior vena cava, which merge in the right atrium and preferentially directed to the right ventricle [32, 37]. Seventy-five percent of the total right ventricle output is directed to the central circulation, while only a minor fraction is driven to the pulmonary arteries [26, 32]. The right ventricle communicates with the aorta through the ductus arteriosus. Although the ductus arteriosus is considered the traditional shunt, the shunting phenomenon occurs at the aortic isthmus, located between the latter and the left subclavian artery [24, 26, 39].

Diastolic blood flow is then directed to the thoracic and abdominal descending aorta due to lower vascular impedance in the subdiaphragmatic circulation when compared to the brachiocephalic circulation [25, 39]. The blood oxygen saturation in the descending aorta is around 60%, supplying the lower fetal body through the iliac vessels [26]. However, a portion of the total blood flow is directed to the placenta, through the internal iliac and umbilical arteries, reaching the capillary nets of the placental villi [32, 40]. Therefore, establishing low vascular impedance at the placental level is vital for driving blood flow along the umbilical arteries, thereby establishing fetal-placental circulation [24]. At term, the placenta contains approximately 33% of the total fetal blood volume [25].

## 4 DOPPLER ULTRASOUND

Invasive techniques, such as plethysmography and electromagnetic flowmeters, have been used in preliminary studies to assess fetal condition [41]. However, these studies were conducted under conditions that do not accurately represent the typical intrauterine environment, making their findings irrelevant to clinical practice [41].

The application of Doppler ultrasound has revolutionized medical practice by providing indirect and noninvasive means of evaluating fetoplacental circulation, thereby assessing fetal well-being [42]. The first trials of Doppler ultrasound in obstetrics took place in 1977, enabling the detection of blood flow in the umbilical cord using spectral Doppler [42, 43]. McCallum *et al.* obtained the first wave spectrum of the umbilical arteries postpartum, while Fitzgerald's research group was able to study the umbilical wave spectrum *in utero* [4, 43-45]. The introduction of color and pulsed-wave Doppler allowed for the identification of specific fetal vessels in the central circulation and the establishment of velocity waveforms as early as 7 weeks of gestation [42-45]. So, in the 1980s, Gill *et al.* quantified blood flow in the fetal intra-abdominal umbilical vein, and Eik-Nes *et al.* estimated quantitative blood flow in the descending thoracic aorta by using a probe associated with a pulsed-wave Doppler transducer, ensuring proper insonation angles in the targeted vessels [4, 43].

Doppler ultrasound has been employed to study the FVW in intracerebral and umbilical arteries, as well as the fetal descending aorta [5]. In the fetal descending aorta, the FVW is characterized by an acceleration time that reflects myocardial contractility, a peak systolic velocity (PSV) that corresponds to the maximum velocity in the aorta during ejection, and a deceleration phase comprising fast and slow components. The slow component exhibits a post-systolic notch and an end-diastolic flow velocity (EDFV). The post-systolic notch corresponds to the closure of the aortic valve during early diastole, while the EDFV represents the lowest velocity reached during diastole, which indicates peripheral vascular resistance (PVR) [1, 4, 6].

The FVW of umbilical arteries is similar to that of the aortic spectrum; however, it lacks the post-systolic notch, has a lower PSV, and has a higher EDFV compared to the aortic waveform [1]. Several physiological factors influence the FVW of the aorta and umbilical arteries, including cardiac contractility, vessel diameter and compliance, distance from the heart and placenta to the measurement site, blood viscosity, and PVR [4, 46]. The total PVR in the fetus is a result of the vascular impedance of both the systemic and placental circulations [7]. Placental vascular resistance is dependent on the number of functional placental tertiary villi [42]. Research studies have reported Doppler abnormalities when at least 30% of the terminal villous arteries are obliterated, and absent or reversed EDFV occurs when 70% of the placental vascular bed is abnormal [7, 42]. Furthermore, Doppler findings in high-risk pregnancies may emerge before changes are observed in fetal non-stress testing, emphasizing the value of evaluating Doppler waveforms in obstetrical practice [42, 47].

As vascular impedance cannot be directly measured, Doppler indices are assessed noninvasively using pulsed-wave Doppler [1,7,48]. Numerous Doppler indices have been used in clinical research to identify fetuses at an increased risk of perinatal morbidity and mortality, thereby guiding the need for closer fetomaternal surveillance [47, 49]. For instance, to distinguish between normal and growth-restricted fetuses, Thompson *et al.* (1986) demonstrated that indices reflecting downstream impedance were preferable [50, 51]. Among these, the systolic/diastolic (S/D) ratio, pulsatility index (PI), and resistance index (RI) are commonly used and have shown reproducibility [41, 47]. As mentioned earlier, the PSV and EDFV are two critical points in the analysis of FVW, representing the highest and lowest velocities achieved during systole and diastole, respectively [24]. The S/D ratio, or A/B ratio, is obtained by dividing these two indices and reflects placental resistance [42, 52]. The PI is calculated by dividing the PSV minus EDFV by the time-averaged maximum velocity (TAMX) over a cardiac cycle, while the RI corresponds to the difference between PSV and EDFV, divided by the PSV [51-53]. Although highly correlated, the PI is considered the best index for estimating the characteristics of the arterial waveform compared to the RI or S/D ratio [53]. This may be due to the fact that the PI does not rely on the presence of EDFV and demonstrates a linear correlation with vascular resistance [53]. In contrast, both RI and S/D ratios follow a parabolic curve as vascular resistance increases, approaching infinite if EDFV is absent or reversed [53, 54].

The clinical application of Doppler indices relies on the availability of reference ranges for arterial vessel waveforms [47]. In the past, these reference ranges were derived from cross-sectional studies or had methodological limitations, such as small sample sizes and inadequate study designs, limiting their clinical usefulness [47]. Longitudinal studies have been conducted to improve the accuracy and reproducibility of these measurements [47, 54]. Certain technical aspects of Doppler acquisition have also been considered, including recording Doppler during fetal quiescence, using color Doppler to identify the specific vessel of interest, and optimizing gain, pulsed-wave frequency, and insonation angle [50, 54]. While some Doppler indices can be obtained independently of the insonation angle, such as the PI and the RI, an insonation angle below 30º maximizes the Doppler shift and facilitates obtaining a better arterial FVW [42, 54, 55]. Furthermore, the accurate evaluation of Doppler indices depends on selecting the appropriate sampling point within a vessel, which varies according to the gestational age [54, 55].

Given the importance and practical utility of umbilical Doppler in assessing fetal growth and well-being, there is a wide range of publications focusing on changes in FVW and impedance reference ranges in low-risk pregnancies, particularly in the late-second and third trimesters [48, 49]. Concerning the fetal descending aorta, substantial research is emerging regarding its hemodynamic Doppler profile, as it appears to be closely associated with cardiac performance and the presence of fetal pathology [56-58].

### 4.1 Doppler Ultrasound in Low-risk Pregnancies

#### 4.1.1 Fetal Descending Aorta

Animal experiments have demonstrated that approximately 50% of the total cardiac output is distributed to the lower fetal body and placenta through the fetal aorta [3,59]. Consequently, evaluating the fetal aortic FVW could provide valuable information about fetal and placental hemodynamics. It is also considered a crucial indicator of proper development and growth of the fetal cardiovascular system [1, 2, 6, 57]. Similar to umbilical Doppler, the assessment of aortic Doppler has shown increasing interest in evaluating fetal well-being, correlating with pregnancy complications, such as FGR, anemia, and hypoxia [6]. However, the utility of fetal aortic FVW in clinical practice has been precluded by methodological issues, notably the large Doppler insonation angle later in pregnancy, or even the dorsoanterior position of the fetus, limiting its application to investigational purposes [53, 56].

The literature is still scarce regarding reference ranges for the fetal descending aorta [56]. The aortic FVW is characterized by low EDFV and a more prominent postsystolic notch throughout pregnancy, resulting from an increase in myocardial contractility [3, 6, 56]. When comparing the umbilical and aortic FVWs, notable differences can be observed. The umbilical EDFV is absent until 10 to 12 weeks of gestation, whereas the aortic EDFV becomes positive later in gestation, around 14 weeks [44, 45, 60]. In terms of aortic PI, available data suggest a trend similar to the umbilical PI, showing a decrease until 16 weeks of gestation, although studies present conflicting findings beyond this gestational age [2]. Some studies have suggested a modest increase in aortic PI from 1.79 to 1.95 between 18 to 41 weeks of gestation, while others have proposed a constant PI throughout the second half of pregnancy [3, 4, 46, 56, 61]. Despite a decreased umbilical PI as pregnancy progresses, a constant aortic PI and an increasingly pulsatile waveform of the descending aorta could be explained by compensatory vasoconstrictive mechanisms in major aortic branches, notably renal and mesenteric vascular beds [3]. The aortic PI also depends on fetal behavior and exhibits a negative correlation with the fetal heart rate (FHR), resulting in poorer reproducibility compared to umbilical Doppler [46]. In addition to the aortic PI, the RI follows a similar pattern, with a significant increase between the first and second and the first and third trimesters [6]. The S/D ratio remains constant as gestation progresses [4, 56].

The blood flow in the aorta increases with gestational age, being proportional to the aortic cross-sectional area and fetal body growth [61]. At term, the calculated aortic flow is nearly 500 ml/min, approximately twice the flow in the umbilical artery at 40 weeks [1]. Research studies assessing blood velocity in the descending aorta have reported a mean aortic velocity of 27-32 cm/sec. However, the variation in PSV, especially in the last trimester of pregnancy, remains uncertain [1, 3, 55]. While some authors argue for a progressive increase in maximum flow velocity until 39 weeks of gestation, others have observed an increase from 10 to 32 gestational weeks, reaching a plateau during the third trimester [3, 46, 55, 56, 62]. Considering the fetal descending aorta and the umbilical artery as a single arterial column, a more significant increase of EDFV and a reduction of PSV are observed on the umbilical side throughout gestation Fig. (**2**). This finding suggests significant placental compliance and an adaptation of aortic impedance to accommodate increased cardiac output [1].

**Fig. (3):**
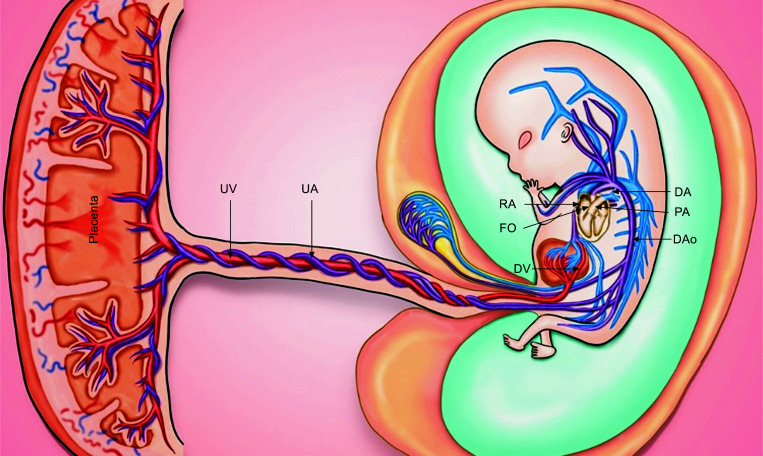
The fetoplacental circulation, highlighting the concept of aortoumbilical column. This arterial column represents the continuation of the fetal descending aorta with the umbilical arteries, draining about 50% of the blood flow of the fetus into the placenta. Limited data have shown that both aortic and umbilical arteries present the same impedance variations, notably a decrease of PI and RI, and an increase of EDFV, as the evaluation point of each vessel becomes distal. By considering the aortic and umbilical arteries as a single arterial column, the aortoumbilical column, it is possible to assess and analyze the FVW and impedance characteristics along its length. This concept suggests that evaluating the aortoumbilical column as a whole may provide valuable information about fetal hemodynamics and potentially identify high-risk pregnancies. **Abbreviation:** DA, Ductus arteriosus; DAo, Dorsal aorta; DV, Ductus venosus; FO, Foramen ovale; PA, Pulmonary artery UA, Umbilical artery; UV, Umbilical vein.

Measuring Doppler indices in the fetal descending aorta may be challenging since its blood flow originates from the cardiac output of both ventricles and is distributed through multiple major arterial branches [55, 63]. However, the distance from the sampling point to the heart has also been shown to impact the aortic FVW [54, 64]. For instance, Bahlmann *et al*. (2001) found a decrease in Doppler resistance indices between the aortic arch (PI 2.34, RI 0.87) and the abdominal aorta (PI 1.87, RI 0.79) [56]. Subsequently, Nishihara *et al.* (2006) confirmed these findings by evaluating Doppler indices at three sampling points (thoracic, under the diaphragm, and abdominal, just below the origin of the renal arteries) [55]. This suggests lower impedance in the abdominal aorta and supports the use of standardized measurements along the aorta to ensure optimal reproducibility of the aortic Doppler indices (Fig. **3**) [55].

#### 4.1.2 Umbilical Arteries

Despite using Doppler ultrasound to assess umbilical circulation during the second and third trimesters of pregnancy, its application in the first trimester and its clinical significance remain unknown [54, 65].

Early studies have established the pattern of umbilical impedance in uncomplicated pregnancies, showing a decrease in flow impedance as pregnancy progresses [1, 3, 42, 44, 47]. Changes in umbilical FVW can be observed as early as the embryonic stage, around 5 to 6 weeks of gestation, when the uteroplacental circulation begins to develop [44]. The umbilical flow is characterized by an absent EDFV at early gestation and, consequently, higher vascular resistance in fetoplacental circulation [1, 19, 60, 66]. The timing of the transition from absent to positive EDFV varies between studies, likely due to population heterogeneity and methodological differences. For example, Arduini *et al.* (1991) reported the detection of almost 20% of positive diastolic velocities around 10 weeks of gestation, while Mercé *et al.* (1996) and Van Zalen-Sprock *et al.* (1994) did not detect positive EDFV until 11 to 12 weeks of gestation [44, 60, 66]. Positive EDFV is consistently identified between 14 to 15 weeks of gestation, which can be attributed to the second wave of the trophoblastic invasion of spiral arteries [1, 44, 67]. This invasion leads to a progressive reduction in vascular resistance in uteroplacental vessels, along with the development of the cardiac area and placental bed throughout gestation [1, 44, 47, 65]. Earlier studies using electromagnetic flowmeters and radionuclide-labeled microspheres have demonstrated an increase in umbilical artery flow from 8.5 mL/min to 30 mL/min between 12 and 19 gestational weeks, corresponding to an increase in cardiac output directed to the umbilical vessels from 17% to 33% [68, 69].

Regarding other Doppler indices, a gradual decrease in PI and RI values, as well as an increase in PSV have been reported since the early-second trimester, particularly from 16 weeks of gestation [3, 47, 66, 69] To identify the abnormal progression of gestation, reference ranges of umbilical Doppler indices have been established starting at 19 weeks of gestation, based on 130 low-risk pregnancies [47]. Despite a weekly reduction of PI and RI of up to 0.01 and 0.005, respectively, this workgroup also demonstrated a negative association with both placental and birth weights [47]. Therefore, after adjusting to gestational age, a further decrease in both PI and RI was observed for every additional 100 grams in placental weight and 500 grams in birthweight [66]. Placental vascular resistance decreases between the second and third trimesters, from 0.65 to 0.15 mmHg/mL/min, respectively, while EDVF increases and the S/D ratio progressively decreases [1, 7].

In the first trimester, however, umbilical Doppler indices are incongruous. Mercé *et al.* (1996) have reported changes in umbilical PSV and RI similar to those observed in the second half of pregnancy when evaluating uteroplacental circulation between 4 to 5 weeks of gestation [60]. However, significant changes in umbilical PI have not been consistently observed, unlike later in pregnancy where a decrease in PI is noted [40, 44, 60, 66]. This discrepancy may be due to errors in calculating Doppler indices at the embryonic stage when recorded velocities are relatively low [60]. Additionally, there is inconsistency regarding the progression of RI and the variability of umbilical PI during the first trimester [46, 60, 66, 70].

Preliminary studies have suggested that umbilical FVW may differ depending on the sampling points within the umbilical cord [54, 71-73]. Studies evaluating Doppler indices in the second and third trimesters have shown a decreasing pattern of PSV and resistance indices from the proximal umbilical artery to its placental insertion (Fig. **3**) [71-73]. A correlation coefficient of -0.45 was calculated when comparing the S/D ratios between these sampling points, indicating a mean difference at 20 weeks higher than 1. In contrast, no difference was found between the two sampling points at 38 weeks [72]. Conversely, to early reports, Mine *et al.* (2001) compared Doppler indices of both umbilical arteries at the same points to exclude possible impedance differences [71-73]. Still, no significant differences were found in assessing umbilical PI during the first trimester at three different sampling points (fetal insertion of the umbilical cord, free loop, and placental insertion) [73]. Since the umbilical cord is only a few millimeters long during the first trimester (compared to 44-80 centimeters at full term), its length does not affect Doppler indices. This is because the shorter the vessel, the smaller the impedance differences between its ends [54, 74]. Additionally, the FHR can contribute to Doppler variability in the second half of pregnancy. Nonetheless, in the first trimester, the maturation of the parasympathetic nervous system is not yet fully established, making Doppler indices independent of FHR [54]. So, Martinez *et al.* (1995) concluded that in the absence of EDFV in early gestation, PI remains constant and serves as the best index to characterize umbilical FVW in the first trimester, without the need for adjustments to sampling points along the umbilical cord [54].

### 4.2 Doppler Changes in High-Risk Pregnancies

#### 4.2.1 Fetal Descending Aorta

Several studies have demonstrated significant changes in fetal aortic impedance in pregnancies complicated by FGR [4, 43, 46]. Griffin *et al.* (1984) observed an increase in mean aortic PI in fetuses with FGR, with 75% of them exhibiting a PI two-standard deviations (SD) above the normal range [4]. This finding was justified by decreased PSV and decreased or absent EDFV, particularly in hypertensive women [4, 46]. When FGR and fetal distress occur concomitantly, the aortic FVW showed alterations 2 to 3 weeks before a nonreassuring nonstress test (NST), indicating a correlation with low umbilical vein pH [4]. This suggests that changes in the aortic FVW emerge earlier in pregnancy compared to cardiotocographic changes, which are only present when hypoxia is established [4]. However, the optimal timing for intervention when abnormal aortic FVW is detected remains unknown, as there is no established correlation between aortic FVW and umbilical vein pH [4]. Additionally, there have been correlations between mean fetal aortic velocity and blood gas parameters such as hypercapnia, acidosis, and hyperlacticaemia [75]. Therefore, assessing fetal aortic impedance may be beneficial in cases of hypoxia, as decreases in EDFV in the fetal descending aorta may precede hemodynamic changes in the umbilical artery [1, 76]. Furthermore, Cahill *et al.* (2021) found that in cases of discordant PI between the two umbilical arteries, the aortic PI tends to align with the more normal umbilical PI, highlighting its potential utility in situations where one of the umbilical vessels shows abnormal PI, to avoid false-positive predictions of adverse perinatal outcomes [77, 78].

In pregnancies complicated by gestational hypertensive disorders, women with chronic and stable hypertension generally present similar antenatal and postnatal outcomes compared to normotensive women [6, 62]. Furthermore, Joupilla *et al.* (1986) found no differences in aortic FVW between low-risk pregnancies and those complicated by chronic hypertension [62]. However, hypertensive women diagnosed with FGR showed higher impedance indices along the fetal descending aorta [62]. More recently, a comparison between low-risk pregnancies and hypertensive women throughout gestation revealed lower aortic RI and PI in hypertensive women [6]. Additionally, aortic PI showed significant differences between low and high-risk pregnancies in the second and third trimesters, while aortic RI showed differences only at baseline [6]. The increased PVR observed in women with unstable hypertension may be attributed to higher levels of vasoactive agents crossing the placenta, leading to fetal hemodynamic changes, particularly in the fetal aorta [6]. These vasoactive agents may limit the increase in aortic PI, resulting in a progressive rise that is less pronounced compared to uncomplicated pregnancies [6]. In addition, by exhibiting a circulatory system independent from the mother's, the fetus may develop adaptive mechanisms to get protected from maternal hemodynamic compromise [6, 62, 79].

The application of Doppler ultrasound in pregnancies complicated by red blood cell alloimmunization aims to detect hemodynamical changes that could serve as reproducible markers of significant fetal hemolysis [80-82]. In the 1990s, Nicolaides *et al.* observed a positive correlation between fetal hemoglobin (Hb) deficit and mean fetal aortic velocity [80]. However, in the presence of fetal hydrops, a negative correlation was found, suggesting a decrease in aortic blood flow velocity as anemia worsens [80]. These findings indicate that while fetuses can tolerate mild to moderate anemias, severe anemia can lead to cardiac decompensation (due to hypoxia and hyperlacticaemia), blood flow redistribution, and increased PVR, as a result of clogged placental capillaries by circulating erythroblasts [80]. Early diagnosis is crucial as it allows for *in utero* transfusions to be performed before the development of fetal hydrops [80-82]. The mortality rate for hydropic fetuses undergoing transfusion was found to be 31%, indicating an inability of the fetal heart to cope with the additional volume load [82]. Although not currently established as a screening method, pioneer studies of aortic Doppler have shown good positive predictive value, as well as high sensitivity and specificity, suggesting its potential in predicting fetal anemia [82].

#### 4.2.2 Umbilical Arteries

The application of umbilical Doppler ultrasound in the second half of pregnancy is well-established in clinical practice [83]. Meta-analysis and randomized controlled trial studies have primarily focused on immediate and intermediate perinatal outcomes, demonstrating that abnormal umbilical FVW serves as a predictor of low Apgar score, nonreassuring fetal status, the presence of thick meconium and admission to the neonatal intensive care unit (NICU) [84, 85]. Furthermore, its use in high-risk pregnancies has been associated with a 38% reduction in perinatal mortality [86, 87]. However, its utility in uncomplicated pregnancies remains controversial and in high-income countries is not recommended [77, 88]. Some studies have investigated the application of umbilical Doppler in healthy pregnancies to predict moderate to severe small-for-gestational-age (SGA) fetuses, but no association with gestational hypertensive disorders has been found [89]. Goffinet *et al.* (1997) demonstrated a continuous relationship between RI and birthweight, showing a reduction of 462 grams in birthweight when the RI value changed from 0 to 1 [90]. Moreover, in low-income countries, increased RI rates of 13% and absent EDFV rates of 1.2 to 1.5% have been identified, presenting an association with SGA, NICU admissions, and perinatal deaths [77]. Impedance indices in these settings are approximately ten times higher than in high-income countries, suggesting that fetal umbilical Doppler could be considered a screening tool in low to middle-income countries [88-90].

Early studies have established that higher impedance indices are associated with numerous gestational complications [44, 65, 83, 91]. Friedman *et al.* (1989) reported an abnormal umbilical S/D ratio in 24% of high-risk pregnancies (including hypertensive disorders, FGR, and women with systemic lupus erythematosus). Rochelson *et al.* (1989) found absent EDFV in 82% of growth-retarded fetuses and 63% of fetuses with fetal distress [58, 92]. Arduini *et al.* (1991) also found an association between FGR and hypertensive disorders with abnormal umbilical PI in the third trimester, although the PI values of these fetuses fell within the normal range during early gestation [44]. It has been hypothesized that insufficient placental angiogenesis or obliterative processes of small muscular arterioles beyond the first trimester may explain the normal impedance observed during the first half of pregnancy [91, 93]. Cases of SGA neonates with abnormal umbilical artery FVW also have shown higher rates of maternal hypertensive disorders, indicating a correlation with the development of placental ischemic disease [77, 94]. In fetuses with early-onset FGR, delivery may occur 5 weeks earlier if EDVF is absent, being more likely to experience fetal distress and preterm birth [94, 95]. Despite having a lower birthweight, long-term neurodevelopmental outcomes have shown inconsistent results in the literature, ranging from equal to poorer outcomes when compared to fetuses with positive EDFV [94, 95].

Fetuses affected by major aneuploidies, such as trisomy 21, may exhibit fetal and placental growth dysfunction [94]. This phenomenon is attributed to anomalies in trophoblast proliferation, resulting in placental vascular insufficiency, which is also present in pregnancies complicated by FGR and preeclampsia [64, 93, 96]. Moreover, as placental vascular resistance contributes to fetal cardiac afterload, it is increased in impaired placentation, leading to cardiac failure and increased nuchal translucency (NT) in fetuses with coexisting heart defects [64, 93, 97]. Numerous studies have proposed a potential link between umbilical PI, NT thickness, and chromosomal abnormalities during the first trimester. However, the existing data on this topic is inconsistent and conflicting [93, 96-99]. In 1996, a prospective study reported an increased umbilical artery PI in 66% of 12 cases of trisomy 21 and 54% of all detected chromosomal defects [91]. Martinez *et al.* (1997) assessed the impact of using NT and umbilical Doppler as screening tools for aneuploidies, concluding that women showing both NT and umbilical PI within normal ranges had a 0.6% probability of having an affected child [99]. This probability increased to 23% if either parameter was altered and rose to 75% when both parameters were increased [99]. Umbilical PI also shown to increase from the early-second (33%) to the late-third trimester of pregnancy (64%) [97]. However, Brown *et al.* (1998) and Borrel *et al.* (2001) found no correlation between the 95^th^ percentile of umbilical PI or reversed EDFV and aneuploidies [64,100].

In conclusion, the use of the umbilical Doppler in gestational hypertensive disorders and FGR is validated in obstetrical practice [100]. However, its application in certain clinical conditions, such as pregestational or gestational diabetes, or as a stand-alone screening method for aneuploidies in the first trimester, remains of questionable value [100, 101].

## 5 DERIVATION OF REFERENCE CURVES IN OBSTETRICS

In obstetrics, reference charts for fetal variables, including biometric measurements and Doppler impedance of major vessels, play an essential role in assessing fetal development based on gestational age (GA) [7, 102]. Prospective studies are recommended for deriving centiles in order to minimize sample bias and ensure accurate reference chart construction [102, 103]. Reference curves can be derived from cross-sectional data (a single measurement from each fetus) or longitudinal data (multiple measurements per fetus at different time points). Cross-sectional data is preferred for developing reference centiles for fetal size [104, 105].

The sample size is an important consideration in reference chart construction. A larger sample size leads to more precise estimates. Special attention should be given to the distribution tails as they identify extreme centiles based on limited observations. Large sample size is desirable to estimate the centiles accurately, but it should account for excluded cases due to clinical complications, early deliveries, or drop-offs from the study [104]. Sample size calculations depend on the statistical method used in the analysis. For regression-based reference ranges, formulae developed by Royston and extended by Bellera and Hanley in 2007 are commonly used [106-108]. For data following a conditional normal distribution, the formula for the standard error of the pth centile is:


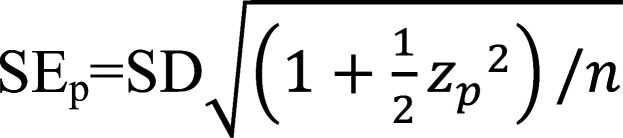


where **SD** is the standard deviation of the measurement; **z_p_** is the pth centile of the standard normal distribution and ***n*** is the sample size.

Parametric and non-parametric (empirical) statistical methods can be used to estimate reference centiles. Reference centiles should exhibit smooth changes with GA and consider the increasing variability among fetuses as gestation progresses [103]. The “mean and SD model” is a straightforward parametric approach that has shown promising results in identifying reference centiles [109, 110]. It assumes that at each GA, data comes from a standard population, and the mean of variance of the fetal variable is modeled as a polynomial function of GA. Z-scores, representing observed values on a standard scale with mean=0 and SD=1, can be used to assess model fit. Fractional polynomials, an extension of the “mean and SD model” introduced by Royston and Altman in 1994, have gained popularity in recent years for constructing centile curves [103].

The goodness-of-fit of a chosen model can be assessed using numerical measures specific to the statistical modeling used, but a graphical approach should always accompany it. A scatterplot of the observations across GA with the calculated centiles superimposed is a simple informative plot that should be included in publications. The proportion of points outside the outer centiles should align with the centiles and be evenly distributed throughout the gestation range [105]. The proportion of observations falling between the fitted centiles may also be consistent. Additional information that should be provided for future users of the reference charts includes a table with selected fitted centile values (*e.g.*, 10^th^, 50^th^, and 90^th^) and, whenever possible, the explicit equation for calculating desired centiles and Z-scores [104].

## 6 AORTOUMBILICAL COLUMN – WHAT DO WE KNOW?

As previously mentioned, the abdominal portion of the descending aorta carries blood flow to the fetal organs as well as to the placenta through the umbilical arteries. This results in a balanced distribution of blood flow between the fetal and placental compartments. Therefore, it is possible to visualize the flow of blood from the fetal aorta into the umbilical arteries by using Color Doppler imaging during early gestational ages.

Several studies have shown that the impedance indices of both the fetal descending aorta and the umbilical arteries undergo similar modifications as pregnancy progresses and along the length of the referred vessels. These findings have prompted the question of whether the fetal descending aorta and umbilical arteries should be treated as separate entities in Doppler assessment or as components of a single arterial vessel whose changes in the hemodynamic patterns could help identify complicated pregnancies at early gestational ages. However, it is important to note that there is currently a lack of studies specifically assessing FVWs of the umbilical and aortic vessels during the first trimester of pregnancy. In addition, there is a lack of published evidence regarding reference charts for impedance indices and the variation of impedance between these two vessels in early gestations.

Regarding the single artery hypothesis, our research group has proposed the term “aortoumbilical column” (Fig. **3**), to describe this “new” vessel, which can be easily identified in first-trimester pregnancies using Color Doppler imaging. Based on the physiology of the fetoplacental unit, in uncomplicated pregnancies, we would expect to observe higher PI and RI values, along with low EDFV at proximal assessment points. As we move along the arterial column towards the placental insertion, we anticipate a progressive decrease in blood flow resistance. These changes could be attributed to the trophoblastic invasion that occurs in the late-first and early-second trimesters, as well as the fetal hemodynamic adaptation to meet the increasing demands for nutrients and oxygen as the fetus develops throughout gestation.

As mentioned earlier, there are several reasons why measuring arterial impedance indices at early gestational ages is preferred. First, the shorter length of the umbilical cord implies minimal variations in impedance between both extremities of the cord. Second, during the first trimester, there is an absence of parasympathetic maturation, which means that Doppler variations are independent of FHR. This allows for a more reliable evaluation of the arterial impedance without confounding effects from FHR variability. Finally, in early pregnancy, the elasticity of blood vessels and increased cardiac output have a minor impact on impedance variations. This facilitates the detection of subtle changes in blood flow resistance along the aortoumbilical column during early gestational ages.

The fetal central circulatory system is particularly sensitive to hemodynamic changes associated with fetal-placental insults. Therefore, changes in Doppler FVW of the aortoumbilical column may serve as an early indicator of fetal deterioration. So, establishing its FVW characteristics and creating reference charts for Doppler indices in low-risk pregnancies could aid in the early recognition of maternal-fetal complications, including FGR, preeclampsia, fetal anemia, and chromosomal abnormalities. In a preliminary assessment conducted over the course of one year, the aortoumbilical column was examined during the first trimester (mean gestational age of 13 weeks) in 115 women with uncomplicated pregnancies. Our findings revealed a trend towards an increase in the mean PI between the fetal descending aorta and the intraabdominal portion of the umbilical arteries, corresponding to mean PI values of 1.72 and 2.24 respectively. Additionally, there was a progressive decrease in blood flow resistance from the intraabdominal portion of the umbilical arteries to the placental cord insertion, with mean values decreasing from 2.24 to 1.68. The increased blood flow resistance at the intraabdominal portion of the umbilical artery may be attributed to the higher impedance of blood circulation directed towards the lower limbs. As mentioned above, approximately 50% of the blood circulation from the fetal descending aorta is directed toward the placental compartment, while the other half reaches the fetal organs and lower limbs. If impedance were to decrease solely along the aortoumbilical column, blood flow would preferentially be directed to the lower resistance compartment, resulting in hypoperfusion of the lower limbs. Therefore, blood resistance increases at the point where the aortoumbilical column intersects with the blood flow circulation of the lower limbs, maintaining the impedance differential between the placental and fetal compartments. This mechanism ensures equal blood flow distribution in both territories. Furthermore, our study also observed changes in the RI and TMAX in the same direction along the aortoumbilical column. However, it is important to note that further data is needed to validate these preliminary findings and draw definitive conclusions.

## CONCLUSION

Knowledge of human cardiovascular embryogenesis and fetal circulation is indeed crucial to understanding the principles of Doppler velocimetry in obstetrics. Doppler ultrasound has revolutionized the assessment of human fetal and placental blood flow, providing valuable insights into the hemodynamic status of the fetus and identifying abnormal vascular patterns that may be associated with significant pregnancy complications.

Most published studies evaluated the umbilical arteries during the second half of pregnancy. Its abnormal FVW is associated with some clinical conditions of placental insufficiency, such as preeclampsia and FGR. The assessment of umbilical artery Doppler has become an established clinical practice in managing high-risk pregnancies. On the other hand, the clinical application of aortic Doppler has not yet been widely recommended, despite growing evidence suggesting a correlation between blood flow in the fetal descending aorta and cardiac performance. The evaluation of the aortic Doppler can provide valuable information about fetal central circulation and may have the potential to detect early hemodynamic changes. However, data on aortic Doppler in the first trimester of pregnancy is still limited, and further research is needed to establish its clinical utility and reference values.

Understanding the FVW of the aortoumbilical column and establishing its reference charts in low-risk pregnancies are fundamental steps in identifying fetal hemodynamic changes and allowing early recognition and prevention of maternal-fetal complications. By incorporating aortoumbilical Doppler assessment into clinical practice, healthcare providers could potentially enhance the management of pregnancies and improve outcomes for both the mother and the fetus.

## FUTURE CONSIDERATIONS

Developing a protocol to determine normal impedance variations along the aortoumbilical column in the first trimester of pregnancy is an important step in establishing reliable reference charts for clinical practice. Several criteria need to be considered to ensure data reproducibility and the applicability of the developed curves to different patient groups. One important aspect is image magnification, which should be standardized to enable accurate measurements. Additionally, choosing an orthogonal plane for measurement, using an appropriate insonation angle, and selecting the sample volume are important criteria that need to be carefully defined and standardized across operators to minimize variability in Doppler measurements. To assess the reproducibility of the aortoumbilical Doppler assessment, it is essential to evaluate both intra-operator and inter-operator variability. Intra-operator variability assesses the consistency of measurements performed by the same operator, while inter-operator variability examines the agreement between measurements obtained by different operators. Our research group is currently assessing intra-operator and inter-operator variability and the reproducibility of aortoumbilical Doppler assessment. By aiming for good sample reproducibility and low inter-operator and intra-operator variability, the developed protocol will provide reliable and consistent measurements. This will enable the creation of the first-trimester reference charts and their application in clinical practice. Overall, the goals of our investigation group, which include identifying complicated gestations in early pregnancy through the application of these reference charts, are important steps towards improving prenatal care and early detection of maternal-fetal complications.

## Figures and Tables

**Fig. (1):**
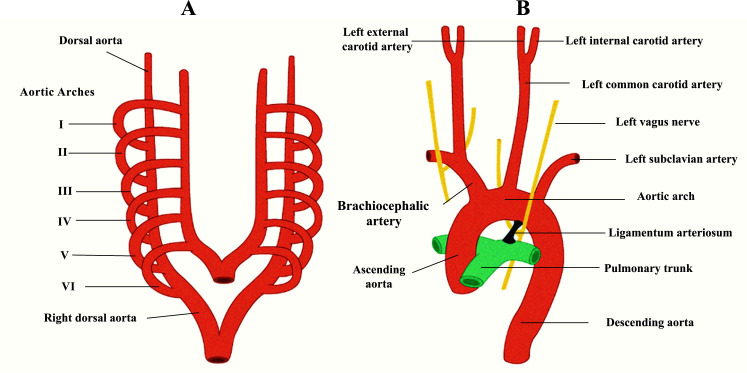
Schematic representation of aortic development during embryogenesis, from the formation of aortic arches and dorsal aorta (**A**) to the maturation of the major vessels in adulthood (**B**) Cells originating from the proximal lateral intraembryonic mesoderm, give rise to the right and left dorsal aorta. These vessels fuse at the midline, caudally to the aortic arches, and connect to the primitive heart through the truncus arteriosus, which is one of the embryological precursors of the ascending aorta. The aortic sac, another embryological precursor, is connected to the dorsal aorta through the aortic arches. Within these arches, pairs I, II, and V regress, while the subsequent arches give rise to specific arterial structures: pair III – the common carotid arteries; pair IV – the segment of the original aortic arch between the left common carotid artery and the ipsilateral subclavian artery; and pair VI – the primary pulmonary trunk. The descending aorta develops from the primary dorsal aorta below the level of T4 [13].

**Fig. (2):**
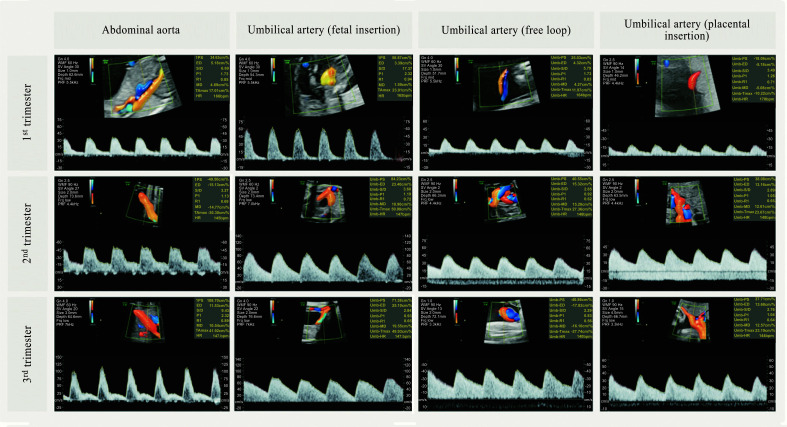
Aortoumbilical column Doppler assessment during pregnancy. From left to right, the evaluation point corresponds to: the fetal descending aorta; umbilical artery, intraabdominal portion; umbilical artery, free loop; umbilical artery, placental insertion. Throughout gestation, there is a noticeable decrease in PSV and an increase in EDFV, resulting in a decrease in impedance along the aortoumbilical column. These changes are more prominent in the first trimester when the trophoblastic invasion is not yet completed, leading to higher impedance indices on the fetal side. Additionally, an increase in impedance can be observed at the intraabdominal portion of the umbilical arteries at the bladder level. This may be attributable to increased resistance in the blood circulation of the lower limbs, maintaining an impedance differential between the low-resistance compartment (placental) and the fetal compartment, allowing blood to be equally distributed to both territories.

## LIST OF ABBREVIATIONS

DV: Ductus Venosus
EDFV: End-Diastolic Flow Velocity
FGR: Fetal Growth Restriction
FHR: Fetal Heart Rate
FVW: Flow Velocity Waveform
GA: Gestational Age
Hb: Hemoglobin
NICU: Neonatal Intensive Care Unit
NT: Nuchal Translucency
PlGF: Placentar Growth Factor
PI: Pulsatility Index
PSV: Peak Systolic Velocity
PVR: Peripheral Vascular Resistance
RI: Resistance Index
SD: Standart Deviation
S/D ratio: Sistole/Diastole Ratio
SGA: Small-for-Gestational-Age
TAMX: Time Average Maximum Velocity
VEGF: Vascular Endothelial Growth Factor
